# A Case of Spontaneous Remission of Membranous Nephropathy After the Removal of Nerve Epidermal Growth Factor-Like 1 Positive Sigmoid Colon Carcinoma

**DOI:** 10.7759/cureus.49892

**Published:** 2023-12-04

**Authors:** Ai Fujii, Norifumi Hayashi, Hideto Fujita, Hitoshi Yokoyama, Kengo Furuichi

**Affiliations:** 1 Nephrology, Kanazawa Medical University, Kanazawa, JPN; 2 Surgery, Kanazawa Medical University, Kanazawa, JPN

**Keywords:** sigmoid tumor, thrombospondin type-1 domain-containing 7a (thsd7a), nerve epidermal growth factor-like 1 (nell-1), nephrotic syndrome, membranous nephropathy

## Abstract

Recently, the association between membranous nephropathy (MN) and malignancy has been recognized in about 30% of epidermal growth factor-like 1 (NELL-1) positive cases. However, the mechanism of association with MN and malignancy remains under search.

In this report, we present a unique case of MN with positive staining for both thrombospondin type-1 domain-containing 7A (THSD7A) and NELL-1. An 80-year-old Japanese woman with nephrotic syndrome (NS) was diagnosed as an immunoglobulin (Ig)G1 subclass predominant secondary MN with weakly positive for THSD7A staining. Then, advanced cancer in the sigmoid colon was found during screening tests for malignancy. After the removal of colon carcinoma, complete remission was achieved at 28 weeks follow-up after operation. Five years later, she remained in remission and passed without recurrence. Thereafter, we examined again newly reported NELL-1 in renal biopsy specimens and found very strong staining along the glomerular capillary walls. Moreover, in resected tumor tissues, NELL-1 was strongly positive at the basal side of adenocarcinoma cells, but THSD7A staining was negative. This case report provides clinical details and highlights the utility of autoantibodies, especially NELL-1, in the diagnosis and treatment of secondary MN with malignancy.

## Introduction

Membranous nephropathy (MN) is a proteinuric glomerulopathy defined by the presence of subepithelial immune complex deposits that induce a spectrum of changes in the glomerular basement membrane (GBM). Further, MN was reported to be associated with the target antigen of the circulating immunoglobulin G (IgG). Recently, thrombospondin type-1 domain-containing 7A (THSD7A) and nerve epidermal growth factor-like 1 (NELL-1) are often identified in the absence of well-known secondary etiologies of MN and also appear to be associated with malignancy in a smaller number of patients [1,2]. In this report, we present a secondary MN case of double positive glomerular staining for THSD7A and NELL-1 with advanced sigmoid colon adenocarcinoma. Only the removal of colon carcinoma, which was staining positive for NELL-1, improved the nephrotic state in complete remission. This case is reported as an important instance associated with NELL-1 and malignancy.

## Case presentation

An 80-year-old Japanese woman visited a previous hospital complaining of proteinuria and edema that had persisted for more than three months. The patient was treated with diuretics and angiotensin receptor blocker (ARB); however, there was no improvement and then she was referred to our hospital in June 2018. Her past medical history was a diagnosis of hyperlipidemia and diabetes mellitus for 20 years. As for her diabetes, she was in good control. Per admission findings, the patient's weight was 65.8 kg, with a weight gain of approximately 8.5 kg with generalized edema. The weight gain and worsening of edema presented gradually. Physical examination revealed mild pallor of the eyelid conjunctiva and bilateral indurated edema of the lower legs. Urinalysis showed 3+ proteinuria and 10.8 g/gCr quantitatively, which gradually increased during three months. The selectivity index of albumin was 0.19. Her serum albumin level decreased to 1.9 g/dL. Then, she was diagnosed with nephrotic syndrome. 

Another laboratory data at admission showed white blood cells 3400 mm^3^, hemoglobin 10.3 g/dL, and hematocrit 30.5%. Other laboratory data showed serum creatinine 0.7 mg/dL, blood urea 10 mg/dL, aspartate aminotransferase (AST) 19 IU, alanine aminotransferase (ALT) 10 IU, creatine phosphokinase (CPK) 31 IU/L, and lactate dehydrogenase (LDH) 214 IU/L (Table 1). Additionally, the immune system showed no hypocomplementemia, and antinuclear antibodies were negative. Infection and tumor markers were also negative, but the fecal occult blood test was positive. Chest X-ray and simple CT showed no significant findings in the chest. Abdominal examination revealed mild atrophy of the kidneys on both sides.

**Table 1:** Changes in laboratory values during the clinical course of two hospitalizations Ht/Hb: Hematocrit/Hemoglobin; PLT: Platelet; SGOT: Serum glutamic-oxaloacetic transaminase; AST: Aspartate aminotransferase; SGPT: Serum glutamate pyruvate transaminase; ALT: Alanine aminotransferase; LDH: Lactate dehydrogenase; CPK: Creatine phosphokinase; ALP: Alkaline phosphatase; γGTP: Gamma-glutamyl transpeptidase; GTT: Glucose tolerance test; CRP: C-reactive protein; U-PCR: Urine protein creatinine ratio.

	Day 1	Day 7	Post-operation Day 40	Day 70	Day 250	After 5 years
WBC (K/μL)	5820	4220	6130	4230	4550	4200
Ht/Hb (%, g/dL)	10.3/30.5	8.5/25.4	9.8/27.9	7.9/24.3	10.8/30.9	11.2/35.4
PLTs (K/μL)	296×10^3^	273×10^3^	282×10^3^	299×10^3^	239×10^3^	182×10^3^
SGOT/AST (U/L)	19	20	29	20	20	12
SGPT/ALT (U/L)	10	9	14	13	19	8
LDH (U/L)	214	240	278	165	198	215
CPK (U/L)	64	60	110	29	81	108
ALP (U/L)	211	181	164	185	253	81
γGTP/GTT (U/L)	10	9	8	10	11	10
Total protein/albumin (gr/dL)	4.7/1.9	3.8/1.5	4.2/1.7	4.2/1.6	6.5/3.7	6.4/3.9
Blood Urea Nitrogen (mg/dL)	10	8	16	9	15	12
Creatinine (mg/dL)	0.7	0.71	0.64	0.71	0.91	0.73
Sodium (mEq/L)	144	145	140	143	137	143
Potassium (mEq/L)	3.7	3.4	3.8	4.1	4	3.7
Calcium(mg/dL)	10.5	10.5	10.5	10.6	9.1	8.9
Phosphorus (mg/dL)	3.8	3.9	non	4.3	3.5	3.5
CRP (mg/dL)	0.04	0.04	0.05	0.03	0.04	0.05
U-PCR(g/gCr)	10.86	4.43	4.38	1.69	0.13	0.15

A kidney biopsy was performed on the second day of admission. Light microscopy findings revealed 24 glomeruli that seemed to be almost normal, except for some glomeruli with mild mesangial changes. Periodic acid-methenamine-silver stain (PAM) staining did not show any clear bubble or spike formation, but Masson's trichrome staining (MT) showed light red staining subepithelial deposits.

**Figure 1:**
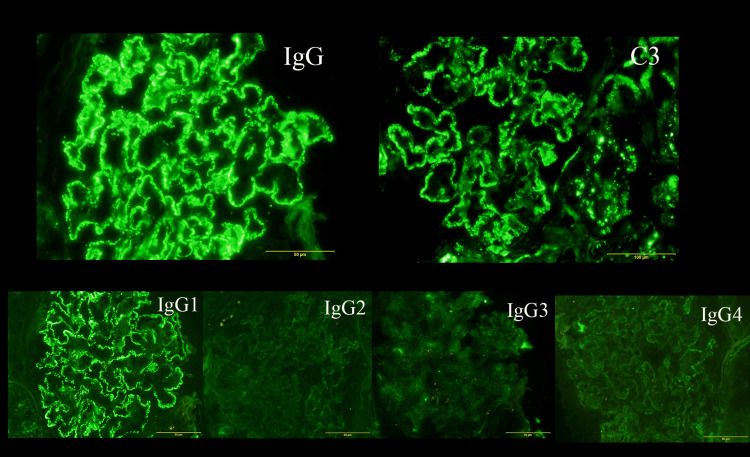
Renal biopsy findings Immunoglobulin (Ig)G and C3 along the glomerular basement membrane (GBM) (immunohistochemical staining, 200X). IgG subclass revealed the depositions of IgG1 (immunohistochemical staining, 400X).

Immunofluorescence study revealed the presence of granular deposits of immunoglobulin (Ig)G and C3 proteins along the glomerular basement membrane. Staining of IgM, IgA, C1q, and anti-phospholipase A2 receptor (PLA2R) were negative. On IgG-subclass staining, IgG1 dominant deposition was observed along the glomerular basement membrane, while IgG2, IgG3, and IgG4 staining were comparatively weak (Figure 1). 

**Figure 2:**
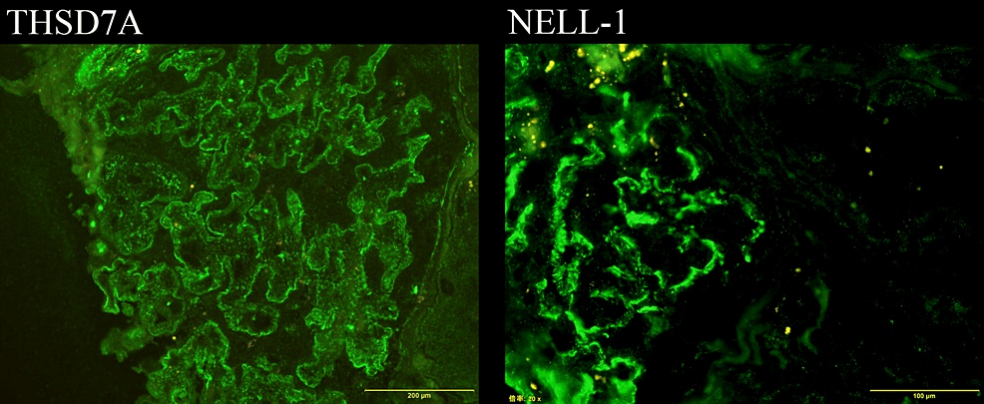
Another immunohistochemical staining Positivity for thrombospondin type-1 domain-containing 7A and nerve epidermal growth factor-like 1 are shown (400X).

Furthermore, THSD7A staining performed in 2018 was weakly positive on the glomerular basement membrane, and NELL-1 staining performed in 2020 was strongly positive (Figure 2). Electron microscopy showed high electron-dense deposits under the epithelium. Based on these results, we have diagnosed stage 1 MN.

Since there have been reports of complications of malignancy in THSD7A stain-positive cases, we suspected that the patient may have secondary MN associated with malignancy. Therefore, we started screening tests for malignancy. The patient had a positive result for occult blood in the stool, and a subsequent lower endoscopy revealed advanced sigmoid colon cancer T3N0M0, Stage Ⅱ per Japanese Society for Cancer of the Colon and Rectum (JSCCR) guidelines. Subsequently, the patient was transferred to the Department of Gastrointestinal Surgery for further evaluation and treatment. When a laparoscopic sigmoid colon resection was performed, the patient's treatment for proteinuria involved only ARB, but no immuno-suppressants.

**Figure 3:**
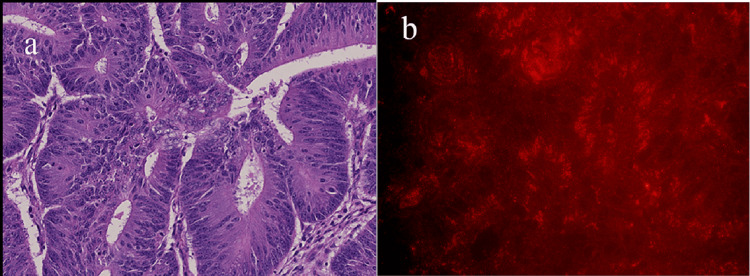
The staining of the section species a: Hematoxylin and Eosin stain (Scar bar = 100μm); b: The tumor tissue stained NELL-1 around the basement of intestine cells and it formed accumulation (immunohistochemical staining, 400X).

The biopsy specimen exhibited high to medium-sized tubular or cribriform structures, leading to the diagnosis of tubular adenocarcinoma. After the resection of advanced colorectal cancer, we performed the staining of THSD7A and NELL-1 for resected specimens. THSD7A was not detected in tumor tissues. On the other hand, NELL-1 was strongly positive at the basal side of adenocarcinoma cells. NELL-1 was also located at the basement membrane part of the normal intestinal tract cells (Figure 3). 

After the resection of sigmoid cancer, her urinary protein excretion decreased and serum albumin improved gradually (Table1). Then complete remission was achieved at 28 weeks follow-up after operation. Five years later, she remained in remission and passed without recurrence.

## Discussion

We reported a rare case of the THSD7A and NELL-1 positive MN associated with advanced sigmoid colon cancer. After the operation, the patient had been in complete remission with conservative treatment without immunosuppressive therapy. This case shows a strong association between colon cancer and MN.

In the past 15 years, many antigens in MN have been identified. The PLA2R and THSD7A are identified as the target podocyte antigen and positive in 70-80% and 1-3% of cases of primary MN, respectively [3]. However, anti-THSD7A antibodies were identified in two of 67 patients with a potential secondary etiology of MN, including systemic lupus erythematosus (SLE) and prostate cancer [4]. Hoxsa et al. showed that eight of 40 patients with THSD7A-positive MN were found to have evidence of malignancy (20%) [5]. A case report showed a patient with THSD7A stain-positive MN associated with gallbladder cancer. The tumor and cancer-infiltrated lymph nodes were observed to be positive for THSD7A staining [6]. On the other hand, Hara et al. reported two THSD7A-associated MN patients in whom cancer was detected at the time of renal biopsy. In these cases, the tumors, which included lung small-cell carcinoma and prostatic adenocarcinoma with neuroendocrine differentiation, were both negative for THSD7A staining [7].

Otherwise, the characterization of the IgG subclass played a significant role in distinguishing between primary and secondary MN and showed an apparent association with malignancy. IgG4 is often found predominant in primary MN. In malignancy-related MN, IgG1 and IgG2 are predominant [8,9]. In the present case, we considered secondary MN associated with malignancy, because IgG1 was the predominant subclass, but not IgG4. Confirmation of IgG subclass and THSD7A staining in MN may be useful in differentiating primary from secondary neoplasms and malignant neoplasm complications, and in determining treatment strategy [10].

Furthermore, our case showed strong positive NELL-1 staining in the glomeruli. Recent studies revealed that NELL-1-associated MN patients frequently linked to malignancies, a higher proportion of malignancy cases observed in the NELL-1-associated group compared to PLA2R- and THSD7A-positive cases of MN. In this case, the glomeruli exhibited positive staining for both THSD7A and NELL-1. However, in the tumor section, THSD7A was found to be negative, while NELL-1 showed positive staining and accumulated around the basement of the intestinal cells. Malignancy found in NELL-1-associated MN included various carcinomas such as prostate cancer, lung cancer, and breast cancer. High NELL-1 expression in these tumors has been observed in Human Protein Atlas data (www.proteinatlas.org/ENSG00000165973) [11]. 

Our data suggested that NELL-1 expression in tumor cells may be associated with the deposition of immune complexes in glomerular capillary walls, implying the occurrence of an antigen-antibody reaction. In addition, as NELL-1 includes a secretory signal peptide, there is a possibility that it activates humoral immunity and stimulates antibody production [12]. However, the limitation of our report is that we did not detect circulating anti-NELL-1 antibodies in this case. Therefore, we were unable to confirm the exact mechanism by which malignancy led to nephropathy. Although NELL-1 is not rarely expressed in tumors, only a few patients will develop NELL-1-associated MN [13]; therefore, further studies are required to determine the triggering events.

Finally, we should search the possible occult malignancy as in this case, especially if they are IgG1 or IgG2 predominant subclass and negative for PLA2R or positive for THSD7A or NELL1. The evaluation should consist of most age-appropriate screening tests and ongoing vigilance will be necessary because occult malignancy may be found later.

## Conclusions

In conclusion, we described the rare case of malignancy associated with THSD7A and NELL-1 positive secondary MN. The treatment of malignancy induced complete remission of the nephrotic syndrome without the use of immunosuppressive drugs, and the patient kept the remission. This case report highlights the utility of IgG subclass and autoantibodies, especially NELL-1, in the diagnosis and treatment of secondary MN with malignancy.
